# Population Structure of *Pseudomonas aeruginosa* from Five Mediterranean Countries: Evidence for Frequent Recombination and Epidemic Occurrence of CC235

**DOI:** 10.1371/journal.pone.0025617

**Published:** 2011-10-03

**Authors:** Makaoui Maatallah, Jihane Cheriaa, Amina Backhrouf, Aina Iversen, Hajo Grundmann, Thuy Do, Philippe Lanotte, Maha Mastouri, Mohamed Salem Elghmati, Fernando Rojo, Snoussi Mejdi, Christian G. Giske

**Affiliations:** 1 Laboratoire d'Analyse, Traitement et Valorisation des Polluants de l'Environnement et des Produits, Faculté de Pharmacie, Monastir, Tunisia; 2 Clinical Microbiology L2:02, MTC-Karolinska Institutet, Karolinska University Hospital Solna, Stockholm, Sweden; 3 University Medical Centre Groningen, Rijksuniversiteit Groningen, Groningen, The Netherlands; 4 National Institute for Public Health and the Environment, Bilthoven, The Netherlands; 5 Infection Research Group, Dental Institute, King's College London and Biomedical Research Centre at Guy's and St Thomas' NHS and Foundation Trust, London, United Kingdom; 6 CHRU de Tours, Service de Bactériologie-Virologie, Hôpital Bretonneau, Tours, France ; Université François Rabelais, Tours, France; 7 Laboratoire de Microbiologie CHU Fattouma Bourguiba, Monastir, Tunisia; 8 Departement of Microbiology and Immunology, Faculty of Pharmacy, University of Alfateh of Tripoli, Tripoli, Libya; 9 Departamento de Biotecnología Microbiana, Centro Nacional de Biotecnología, CSIC, Campus U.A.M., Cantoblanco, Madrid, Spain; University of British Columbia, Canada

## Abstract

Several studies in recent years have provided evidence that *Pseudomonas aeruginosa* has a non-clonal population structure punctuated by highly successful epidemic clones or clonal complexes. The role of recombination in the diversification of *P. aeruginosa* clones has been suggested, but not yet demonstrated using multi-locus sequence typing (MLST). Isolates of *P. aeruginosa* from five Mediterranean countries (n = 141) were subjected to pulsed-field gel electrophoresis (PFGE), serotyping and PCR targeting the virulence genes *exoS* and *exoU*. The occurrence of multi-resistance (≥3 antipseudomonal drugs) was analyzed with disk diffusion according to EUCAST. MLST was performed on a subset of strains (n = 110) most of them had a distinct PFGE variant. MLST data were analyzed with Bionumerics 6.0, using minimal spanning tree (MST) as well as eBURST. Measurement of clonality was assessed by the standardized index of association (I_A_^S^). Evidence of recombination was estimated by ClonalFrame as well as SplitsTree4.0. The MST analysis connected 70 sequence types, among which ST235 was by far the most common. ST235 was very frequently associated with the O11 serotype, and frequently displayed multi-resistance and the virulence genotype *exoS* ^−^/*exoU* ^+^. ClonalFrame linked several groups previously identified by eBURST and MST, and provided insight to the evolutionary events occurring in the population; the recombination/mutation ratio was found to be 8.4. A Neighbor-Net analysis based on the concatenated sequences revealed a complex network, providing evidence of frequent recombination. The index of association when all the strains were considered indicated a freely recombining population. *P. aeruginosa* isolates from the Mediterranean countries display an epidemic population structure, particularly dominated by ST235-O11, which has earlier also been coupled to the spread of ß-lactamases in many countries.

## Introduction

*Pseudomonas aeruginosa* has an extraordinary metabolic versatility, enabling the bacterium to thrive and persist in diverse ecological niches. It is ubiquitously distributed in water, soil, plants, animals and humans, and it is one of the most common nosocomial pathogens in intensive care units (ICUs) [1]. In addition, this opportunistic pathogen is a major cause of morbidity and mortality in cystic fibrosis patients [2].

The pathogenicity of *P. aeruginosa* is conferred by numerous secreted virulence factors. These include elastase, exotoxin A, phospholipase, and protease alkaline [3], [4]. Similar to other gram-negative bacilli, the type III secretion system (TTSS) is considered an important determinant of cytotoxicity and invasion process in which *P. aeruginosa* directly delivers several effector proteins into the cytoplasm of the host cell [5], [6]. Dispersal is also facilitated by the emergence and persistence of multidrug resistant (MDR) clones in hospitals, mainly in intensive care units [7]. The increasing prevalence of MDR organisms is a global health problem [8], because of the limited choice of drugs for clinical treatment. Several studies reported that global dissemination is facilitated by MDR, often belonging to the serotypes O11 [9], [10], [11] and O12 [12], [13], [14], [15].

The sequencing of the whole genome of *P. aeruginosa* PAO1 unveiled one of the largest bacterial genome sequenced, counting 6.3 Mbp and encoding 5,570 open reading frames, the majority of which still have an unknown function. Generally, the size and complexity of the *P. aeruginosa* genome reflects an evolutionary adaptation enabling it to colonize diverse environments and resist a variety of antimicrobial substances [16]. Furthermore, *P. aeruginosa* isolates are known to possess extensive genome plasticity, fluctuating from to 5.2 to 7.1 Mbp [17]. The *P. aeruginosa* genome is a mosaic of a conserved core and variable accessory segments [18], [19]. The core genome is characterized by a conserved synteny of genes, and a low average nucleotide divergence of 0.5. The accessory genome consists of a variable set of genomic islets and genomic islands, most of which belong to an ancient tRNA-integrated island type [20], [21], [22]. This diversity has been a starting point for several attempts of exploring the evolution of this organism and to follow up the global epidemiology.

In a bacterial population, clones are defined as groups of genetically indistinguishable isolates that are asexually descended from a common ancestor [23]. Bacterial population genetics as a discipline has developed over many decades, using *Escherichia coli* as the first model of study [24]. During this investigation the genetic population structure was investigated with multi-locus enzyme electrophoresis (MLEE). This technique aims to detect allelic variation within several metabolic genes simultaneously, on the basis of the differing electrophoretic mobilities of their gene products [25]. This technique has been used for several species [26], [27], [28], [29], [30]. The population structure of most of bacterial species was thought to be clonal [31], [32] until 1993 when Maynard-Smith et al. showed that they could vary from strictly clonal to highly sexual [33].

Multi-locus sequence typing (MLST) is based on the nucleotide sequences of housekeeping genes. Although it can evaluate only the genetic diversity of the core genome it is a robust, standardizable, and portable methodology that can be used in studies of genetic population structures [34], [35] which are facilitated by searchable web-based databases [http://pubmlst.org/paeruginosa]. The MLST database for *P. aeruginosa*, similar to most other MLST databases, is skewed towards isolates displaying particular types of resistance [36], [37], [38], [39], particular infection types (e.g. cystic fibrosis) [40], [41], [42], or particular geographical regions [43], [44] that have been investigated more thoroughly. For this reason available data do not necessarily elucidate population structures as they are prone to phylogenetic discovery bias. Lastly, diversification of *P. aeruginosa* clones has been attributed to frequent recombination, but not comprehensively demonstrated by using MLST-data.

Current evidence suggests that several pathogenic strains belong to epidemic clones that spread over large part of Mediterranean Europe, and that they frequently belong to the O11 and O12 serotypes [45]. However, isolates from the southern side of the Mediterranean basin have not yet been sufficiently characterized. In this present study we analyzed a collection of *P. aeruginosa* isolated from five Mediterranean countries (Tunisia, Libya, Spain, Italy and France) by genotypic and phenotypic methods, including serotyping, antimicrobial susceptibility, virulence gene screening, Pulsed Field Gel electrophoresis (PFGE) and Multi-Locus Sequence Typing (MLST). The aims were to explore the genetic structure of the population, to evaluate the role of recombination in shaping the population structure, and finally to characterize epidemic clones.

## Materials and Methods

### Bacterial strains

Strains were collected from France (n = 30), Italy (n = 6), Spain (n = 20), Libya (n = 25), and Tunisia (n = 60). Isolates were selected to represent various sources to achieve both geographical spread and to elucidate potential relationships between clinical and environmental isolates. *P. aeruginosa* strains were collected from five Mediterranean countries most of which were clinical isolates derived from several sources (Table S1), whereas 18 isolates were environmental strains. We also included *P. aeruginosa* ATCC 27853 and PAO1, as well as the two Clone C strains CSGB8 (clinical) and SG17M (environmental). Strains were identified by standard microbiologic methods such as colony morphology, oxidase reaction, growth at 42°C, and ability to produce characteristic pigmentations on cetrimide agar. A few strains with atypical features were subjected to multiplex PCR targeting the lipoprotein genes *oprI* and *oprL* [46].

### Pulsed-Field Gel Electrophoresis (PFGE)

PFGE typing was performed according to Giske et al [47] with minor modifications. All strains were digested with *SpeI* and the resulting fragments were separated by electrophoresis in 1.2% agarose in a CHEF-Mapper (Bio-Rad, Hercules, USA) in 0.5× Tris-Borate EDTA (TBE) running buffer at 12°C and 6 V/cm for 30 hours with pulse time ranging from 1 to 50 s. *P. aeruginosa* ATCC 27853 was used as reference and included in every 6 lanes to allow calibration and normalization of gels. Gels were stained with ethidium bromide and photographed in a Geldoc EQ (BioRad Laboratories, Hercules, CA). The resulting photographic images were analyzed with the GelCompar II software (Applied Maths, NV St-Martens-Latem, Belgium). The band patterns were compared using the Dice-coefficient by using the unweighted pair group method to determine band similarity accordingly to the criteria established by Tenover et al [48]. A Dice coefficient of ≥0.80 was considered suggestive of possible clonal relatedness.

### Multi-Locus Sequence Typing (MLST)

A total of 110 *P. aeruginosa* strains, most of which contained different *SpeI* macro-restriction profiles, were typed using MLST. MLST was performed according to Curran et al [43] but with slight modifications concerning the annealing temperature of housekeeping gene (*acsA, aroE, guaA, mutL, nuoD, ppsA* and *trpE*) amplification and the designing of new nested sequencing primers for the *acsA* (Forward primer**:** 5-TGT TCG ARG GYG TRC CGA ACT A-3**)** and *nuoD* (forward primer: 5-AAC CAY CCB TCC GCC CAC GG-3) genes. For DNA extraction, overnight cultured *P. aeruginosa* isolates were heated to 100°C for 10 min. Housekeeping genes were amplified by real-time PCR. Reactions were performed on a Rotorgene 6000 (Corbett Robotics Inc; San Francisco, CA, USA) using the QuantiTect SYBR Green PCR mix (Qiagen, Valencia, CA, USA). Amplification reaction mixture comprised 25 µl Quantitect SYBR 2 X Green PCR Mastermix, 1 µM of each primer, template DNA 5 µl and H_2_O to a final volume 50 µl. The PCR program was as follows: 15 min of initial denaturation at 95°C, then 40 cycles at 95°C for 30 s, between 58 to 62°C depending on locus at 30 s, and 72°C for 90 s. A final melting curve analysis was performed to determine the presence or absence of non-specific amplification products. PCR products were purified using Jetquick Spin Column Technique (Genomed GMBH, Löhne, Germany) and used as template for DNA sequencing reaction. Templates were sequenced on both strands with the published primers and the new designed primers using the BigDye Terminator Ready Reaction Mix v3.1. Nucleotide sequences were determined for both strands by ABI Prism 3100 Genetic Analyzer (Applied Biosystems, Foster City, CA). New allelic variants were repeated and confirmed in triplicate.

### Serotyping

Strains were grown overnight on LEC agar at 37°C and subjected to O-antigen serotyping using slide agglutination according to the International Antigenic Typing Scheme (IATS) for *P. aeruginosa* [49]. The serotyping protocol is based on 4 polyclonal and 16 monovalent antisera (Bio-Rad Laboratories, Marnes-La-Coquette, France). Association between serotypes and presence of virulence genes and multidrug-resistance was analyzed with two-tailed Fisher's exact test.

### Detection of exoS and exoU

Virulence gene were detected by PCR amplifications: the reactions were carried out on PTC 200: the 25 µl of volume reaction contained 12.5 µl 2X GoTaq® Green Master Mix (Promega), 0.25 µM of each forward and reverse primer, 2,5 µl of chromosomal DNA and H_2_O to final volume. The PCR program of *exoS* and *exoU* genes was performed as described by Feltman et al [50].

### Antimicrobial susceptibility testing

All isolates were subjected to disk diffusion (Oxoid, Basingstoke, UK) susceptibility testing versus ceftazidime, piperacillin-tazobactam, imipenem, meropenem, ciprofloxacin and gentamicin, according to the guidelines of the European Committee for Antimicrobial Susceptibility Testing (http://www.eucast.org/clinical_breakpoints/; accessed on 18 May 2011). Isolates resistant to ≥three antibiotics from different classes were considered multidrug-resistant (MDR) [51] .

### Analysis of MLST-data

All chromatograms were imported, assembled, edited and trimmed in Bionumerics (6.0: Applied-Maths, Sint Maartens-Latem, Belgium). For each locus, distinct allelic variants were assigned an allelic number and each unique combination of seven allele numbers was assigned a novel sequence type (ST). Based on allelic profiles the evolutionary relationship between isolates was assessed by the algorithm Minimal Spanning Tree (MST) implemented in Bionumerics. The MST is a graphical tool that links the nodes by unique minimal paths in a given dataset, i.e. total summed distance of all branches is minimized [52]. The algorithm uses an ST with the highest numbers of single locus variants (SLVs) as a root node and derives other STs from it. Using a stringent definition of 5/7 shared alleles, MST could then connect all strains and link all related STs into clonal complexes. Accordingly, singletons were defined as STs having at least three allelic mismatches with all other STs.

Descriptive analyses of the genetic variability at MLST loci such as the determination of the mean G+C content, average number of synonymous and non-synonymous sites, average non-synonymous/synonymous ratio (dN/dS), the number of polymorphic sites, the nucleotide diversity per site (π) and the average number of nucleotide differences per site (è) were performed with DnaSPv5 [53]. The software MEGA 4 [54] was used to build a neighbor-joining tree from the concatenated seven sequences using the Kimura-2-parameter distance measure. The eBURSTv3 software (http://eburst.mlst.net) [55], was used also to relate the STs detected in our study to the entire dataset in the MLST database (http://pubmlst.org/paeruginosa/). START2 [56] was used to calculate the index of association (I_A_^S^) between all STs (http://pubmlst.org/software/analysis/start2/). The Neighbor-Net implemented in the software SplitsTree 4.0 [57] with 1,000 bootstrap replicates was used to create the phylogenic network for the individual loci and for concatenated sequences. Further, we used the pairwise homoplasy index (PHI) [58] implemented in SplitsTree 4.0 in order to test the role of past recombination in generating allelic variation. ClonalFrame [59] was used to investigate the population structure by inferring relationship among STs. The basis for this software is a model of genetic diversification that estimates the relative probabilities that a nucleotide is changed as the result of recombination relative to point mutation (r/m ratio). Concatenated sequences were formatted as an eXtended Multi-Fasta Alignment (XMFA). A 50% consensus tree was constructed from 6 runs using the defaults settings. Evidence of recombination events were also searched between sequences of single and concatenated loci using seven algorithms (RDP, Geneconv, BootScan, MaxChi, 3Seq, Chimaera, and SiScan implemented in the RDP 3.27 software [60]. Only recombination events detected by at least three methods and involving parental sequences present in the MLST data set were considered.

## Results

### Antimicrobial susceptibility testing, serotyping and exoS/exoU detection

Antimicrobial susceptibility testing according to EUCAST yielded 52 (35.8%) multidrug resistant (MDR) strains. Most of the MDR isolates were clinical, except three isolates which were environmental: TN310, TN500 and LB30.

Serotyping (Table S1) revealed that serotype O11 with 35.1% frequency was the most prevalent among the isolates (51/145). The other prevalent serotypes were O6 (13.1%), O1 (11%), O4 (8.2%) and O12 (6.2%). Serotypes O3, O10, O5, O7, O9, O2 and O15 were all detected in less than 5% of the isolates. The remaining non-serotypeable strains (9.6%), consisted of 11 polyagglutinable and 3 nonagglutinable isolates. Multidrug-resistance was significantly more common among the O11 isolates (29/51) than among the non-O11 isolates (23/94) (p = 0.0001).

Screening of *exoS* and *exoU* genes was performed in all isolates (Table S1). A total of 61% of strains harbored *exoS* genes, 35% had *exoU*, and 1.3% contained both *exoS* and *exoU*. Among O11 isolates 78% (40/51) had *exoU*, whereas only 12% of the non-O11 isolates (12/94) were positive for *exoU* (p<0.0001). Among the non-O11 isolates *exoS* was more common (83 vs 12%, p<0.0001). Only two strains featured both *exoS*/*exoU* and in five isolates amplification was negative for both genes.

### PFGE

The PFGE analysis was performed on all *P. aeruginosa* isolates except the two Clone C strains. By applying the criteria proposed by Tenover et al [48] for the differentiation of *P. aeruginosa* by pulsed-field gel electrophoresis for possible genetic relatedness (maximum 6 band difference; Dice coefficient 80%). A total of 93 distinct genotypes were recovered from this collection from which 72 strains were singletons and 71 strains segregated into 21 cluster or groups of related isolates comprised of two or more isolates and denoted A–U (Table S1). All these groups displayed close relationship by sharing traits such as MDR profile, serotypes and source of infection or country.

Each strain representative of a distinct profile (Table S1) was subjected to MLST. Occasionally several representatives of a distinct profile were typed with MLST when divergence in serotype, source of infection, geographical origin, MDR profile or genotype of virulence genes was ascertained.

### MLST analysis and phylogenetic relationship

MLST identified 70 STs among 110 strains, most of them with distinct PFGE-profiles (<80% similarity). All selected strains were successfully typed except two strains (excluded from MLST analysis) for which we were not able to amplify the *trpE* locus (strain CECT119), and one strain where both *trpE* and *ppsA* were not successfully amplified (strain FC1). The characteristics of each locus are displayed in Table 1. The G+C content was relatively high, and ranged from 63 (*nuoD*) to 70% (*aroE*) which is similar to the G+C content of the entire genome of the reference strain *P. aeruginosa* PAO1 (67%) [16]. The number of alleles at each locus ranged from 15 to 26. The number of polymorphic sites was overall low and found to be 5.5% for the concatenated sequences, indicating as expected a very low values of π and è (Table 1). The ratio of non-synonymous to synonymous nucleotide changes (dN/dS) was calculated for all 7 gene loci and found equal to 0 for *nuoD* and *trpE* genes but ranged from 0.13% to 9.43% for the remaining genes. A ratio of dN/dS <1 indicates that genes are evolving predominantly by purifying selection.

**Table 1:** Characteristics and polymorphism of housekeeping gene of *Pseudomonas aeruginosa.* π, nucleotide diversity per site; π, average number of nucleotide differences per site; dS: No. of synonymous changes per synonymous site.

Allele	Size	Haplotype	Polymorphicsites	π	π	G+C	dN	dS	dN/dS	PHI test
*acsA*	390	24	21	0.011593	0.010698	0.6879	0.00006	0.04454	0.0013	0.011
*aroE*	495	22	34	0.011903	0.013026	0.7083	0.00325	0.03444	0.0943	0.004
*guaA*	372	26	17	0.007513	0.008666	0.6584	0.00007	0.03001	0.0023	0.036
*mutL*	441	20	22	0.005358	0.009461	0.6705	0.00054	0.01935	0.0279	0.5
*nuoD*	366	15	18	0.004525	0.009327	0.6308	0.00000	0.01879	0.0000	0.018
*ppsA*	369	20	19	0.007714	0.010279	0.6656	0.00020	0.02972	0.0067	0.008
*trpE*	441	24	28	0.009903	0.012901	0.6661	0.00000	0.03671	0.0000	0.001
Concatenate	2874	70	159	0.008504	0.010756	0.6722	0.00067	0.03089	0.0216	0.000

dN: No. of non-synonymous changes per non-synonymous site.

**Figure 1:**
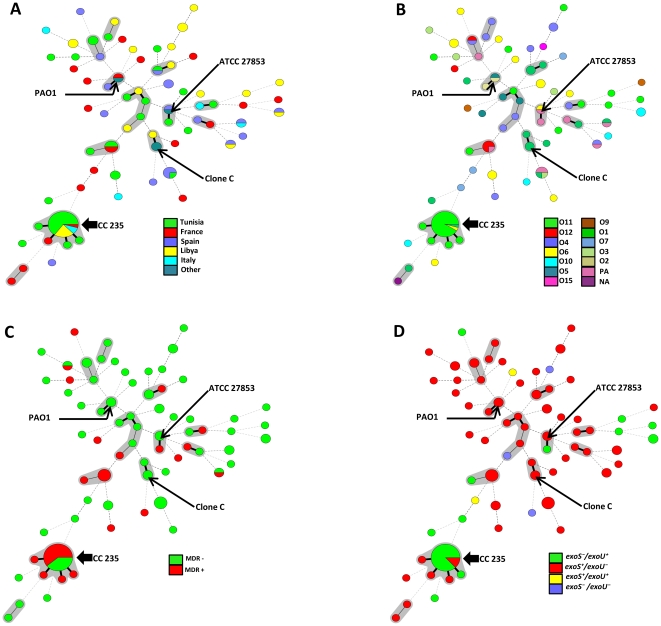
Minimal Spanning Tree (MST) analysis of *Pseudomonas aeruginosa* strains based on MLST data. Each circle corresponds to an ST. The area of each circle corresponds to the number of isolates. The relationships between strains are indicated by the connections between the isolates and the lengths of the branches linking them. Black lines connecting pairs of STs indicate that they differ in one allele (thick lines), two alleles (thin), or three to seven alleles (dashed). Grey zones surround STs that belong to the same clonal complex (clonal complex were defined from this collection, and CC235 was the predominant). Four MST graphs were generated separately based on the following associations. A: ST vs countries, B: ST vs serotype, C: ST vs multidrug-resistance and D: ST vs *exoS*/*exoU*.

In order to determine the clonal relationship between isolates, we used Minimal Spanning Tree (MST) method based on allelic profiles. Two allelic mismatches were allowed for group definition, similar to what is used for group definition with eBURST. The 110 sequence typed isolates were distributed into 50 isolates not belonging to any clonal complexes and 12 groups of a total of 60 isolates corresponding to clonal complexes found in the database (Figure 1). The main clonal complex detected was CC235, consisting of five STs (235, 989, 979, 230 and 227, with ST 235 as the primary founder). The second most frequently encountered clonal complex CC244 consisted of five STs (244, 990, 986, 993 and 654 with ST 244 as the primary found). The 10 others groups or simple complexes were doublets with the following STs linked: 155 and 811, 996 and 242, 992 and 527, 111 and 229, 549 and 699, 228 and 175, 224 and 977, 252 and 984, 17 and 845, 988 and 980.

The MST analysis revealed several interesting relations between countries, serotype, MDR phenotype and presence of virulence genes (Figure 1). MST disclosed the relatedness of STs and displayed a random repartition, especially of the countries and serotypes among isolates. Several STs and some minor clonal complexes were shared by more than one country and one serotype, but most of them were non-MDR and carried the gene *exoS*. Noteworthy, CC235 was identified as a major clonal complex consisting of 27 strains, whereof 25 were serotype O11. Two of the ST235 isolates (TN310 and TN330) were environmental strains. Most of the isolates in ST235 contained the *exoU* virulence gene (n = 20), and 14/27 isolates were MDR.

**Figure 2:**
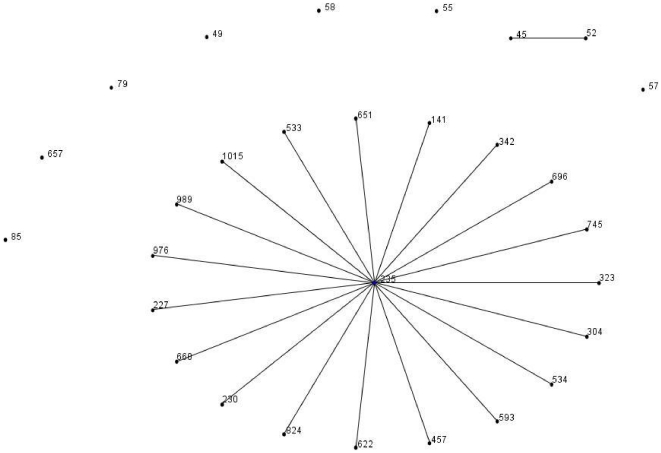
eBURST diagram of the lineage CC235 of all *P.aeruginosa* MLST database displayed like a star-like tree.

By applying eBURST on all *P. aeruginosa* databases, we could demonstrate several clonal lineages. Of them, CC235 consisted of 29 STs whereof ST235 is a primary founder surrounded by 19 SLVs, two double-locus variants (DLV), three triple-locus variant (TLV) and four satellites (>three locus variant; SAT) (Figure 2).

**Figure 3:**
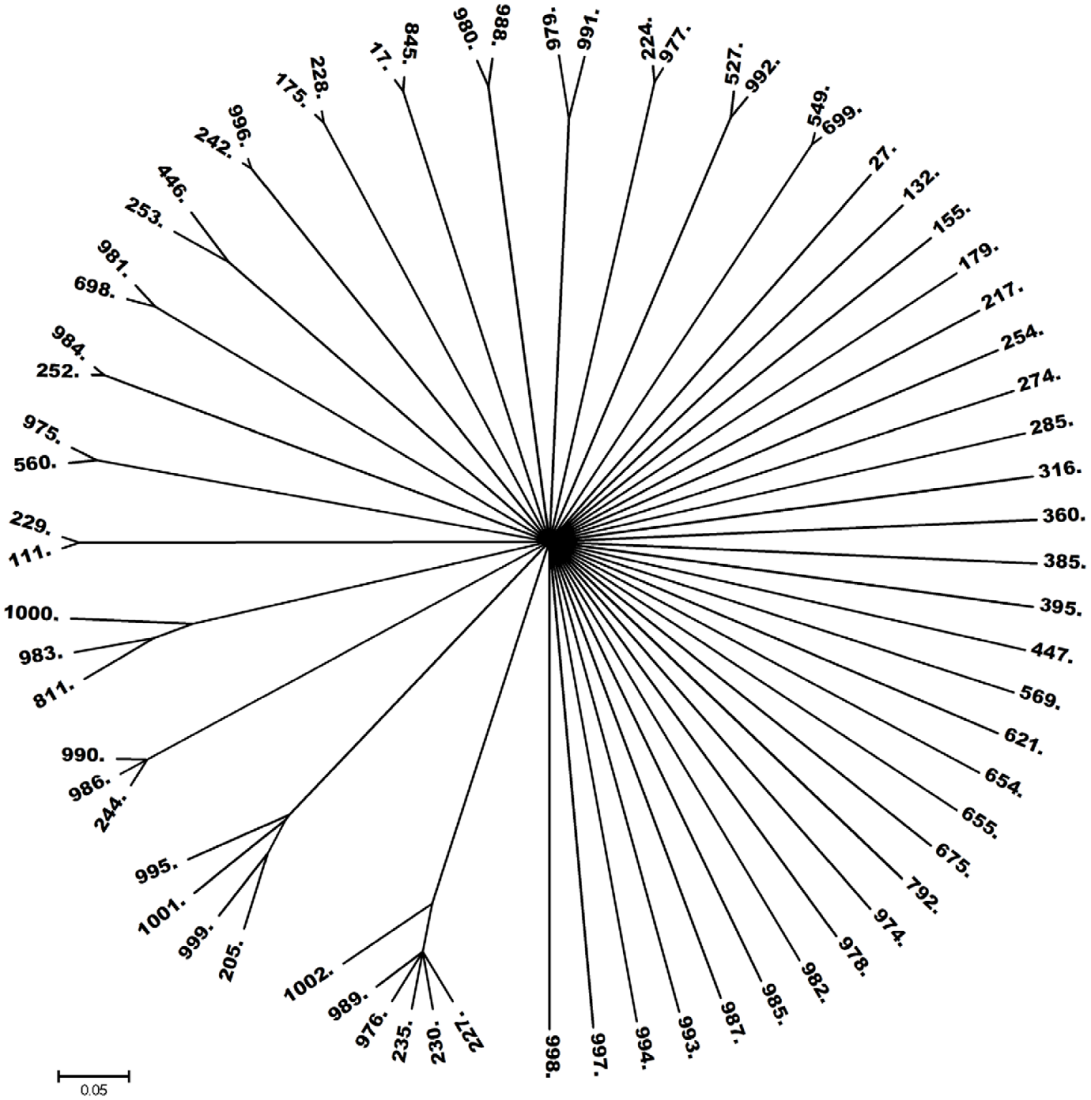
Radial phylogenetic tree of 70 STs of *P. aeruginosa*: A 50% majority consensus tree for the clonal genealogies obtained after six ClonalFrame runs.

Further analysis of the MLST data was conducted with neighbor-joining tree analysis based on concatenated sequences (Figure S1). The analysis revealed a weak bootstrapping value especially with major branches. The dendrogram did not show a clear phylogenetic structure presenting as well defined groups. Instead most of the branches were equidistant with the exception of a few clusters corresponding to clonal complexes previously defined by eBURST. ClonalFrame generated a 50% majority-rule consensus tree from the combination of 6 runs (Figure 3). The resulting dendrogram displays the relationships between STs. Several clusters were identified which were previously obtained by Minimal Spanning Tree (MST) or eBURST.

### Correlation between PFGE and MLST

With PFGE we obtained 93 genotypes or profiles based on a similarity cut-off at 80%. With MLST 70 STs were obtained from 110 strains and 12 clonal complexes were identified. A one-to-one correlation between PFGE patterns and STs existed in 48 cases (Table S1), most of them ST singletons that had unique PFGE patterns (similarity <80%). As expected, several isolates belonging to same PFGE groups (similarity ≥80%) (A, C, E, F, H, I, K, M, O, Q, S and T) were shown to have the same STs (Table S1). Although the correlation was mostly excellent, PFGE groups D (similarity >80%) comprised two isolates displaying different STs. We observed that isolates with identical STs were found in multiple PFGE-types. Seven pairs of strains had respectively the same ST (792, 155, 252, 254. 253 and 111), but all of them had distinct PFGE types. STs 274 comprised 3 isolates, that were all unrelated by PFGE. Interestingly ST 235, identified in 23 strains, was found also in isolates with similarity levels below 80%.

### Evidence of recombination

Based on findings in previous studies [43], [61], [62], [63], [64], [65], [66] we attempted to unravel the evolution and diversity in the population of *P. aeruginosa* from Mediterranean countries. The test of clonality was assessed with a standardized index of association (I_A_^S^), this statistical test attempts to measure the extent of linkage equilibrium within a population by quantifying the amount of recombination among a set of sequences and detecting associations between alleles at different loci. Analysis of the entire dataset of 110 isolates yielded an I_A_^S^ of 0.35 (p< 0.001) and that for the 70 singleton STs was found to be 0.07 (p< 0.001). The obtained values indicate that recombination plays a key role in the distribution of alleles.

**Figure 4:**
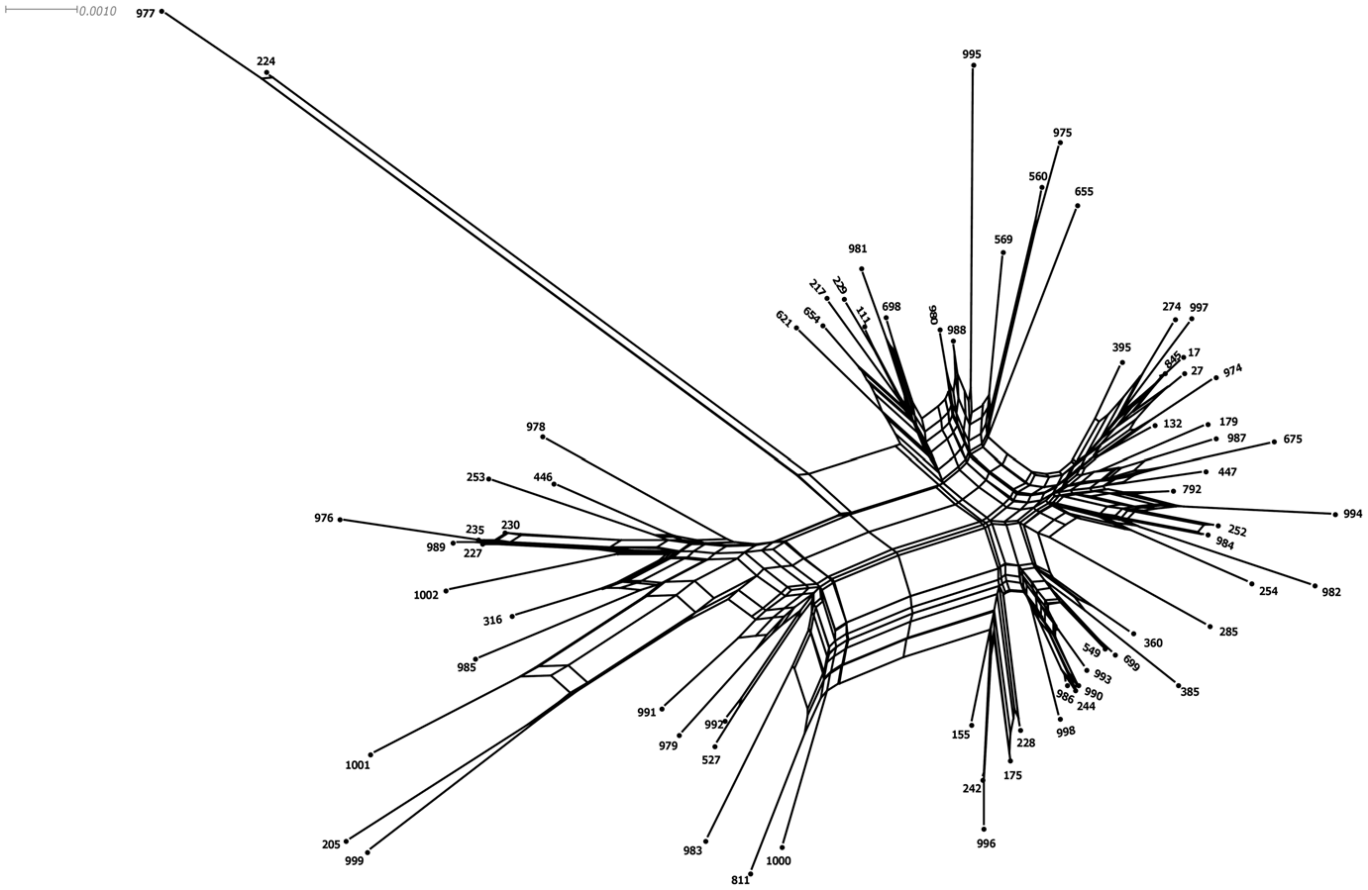
Neighbor-Net graph based on concatenated sequences on 7 housekeeping genes of *Pseudomonas aeruginosa* showing a bushy network structure indicating of a pervasive homologous recombination.

To gather further evidence on the presence of frequent recombination we used SplitsTree v.4 to perform a phylogenetic network analysis with the Neighbor-Net method. This algorithm was conducted separately for each locus (Figure S2) and for the concatenated sequences (Figure 4). The result of PHI test for each locus showed statistically significant recombination, except for the *mutL* allele. Using concatenate sequences, this test produced statistically significant evidence of recombination (p<0.05). Evidence of recombination was also supported by visual inspection of the bushy network structure (Figure 4) with complex parallelogram formation indicative of extensive homologous recombination. In contrast, the Neighbor-Net graph of *mutL* showed a tree-like structure this indicating probably that this gene was not affected by intragenic recombination (Figure S2). Moreover, ClonalFrame confirmed our previous finding and the inferred value of recombination to mutation, r/m, was estimated to be 8.4 (95% CI 4.7–13.7), strongly suggesting that nucleotide change in housekeeping genes occurs more frequently by recombination than *de novo* mutation. Finally, the events of recombination were not accurately identified by the software RDP 3.27.

## Discussion

Several studies suggest that *P. aeruginosa* possesses an epidemic panmictic population structure [67], [68]. The sequencing of 6 loci from 19 clinical and environmental strains revealed a net-like population structure marked by high level of recombination [62]. Lomholt et al, favoured an epidemic structure, consisting of epidemic clones pathogenic in ocular infections with distinct combination of virulence factors [61]. Combined sequence-based techniques, such as sequencing of the outer membrane lipoprotein, with serotyping and pyoverdine type were used by Pirnay et al in a polyphasic approach to reveal extensive genetic mosaicism, particularly in the *oprD* gene [65]. A later study of a large collection of 328 strains from diverse origins and habitats was in accordance with the first one, confirming the non-clonal epidemic population structure of *P. aeruginosa* and indicating that there are no widespread cystic fibrosis epidemic clones [64]. More recently, Curran et al analysed population structure by developing a Multi-Locus Sequence Typing (MLST) scheme and suggested a non-clonal structure punctuated by closely related genotypes or clonal complexes [43]. Furthermore, in 2007 Wiehlmann and colleagues analyzed 240 *P. aeruginosa* strains with a DNA array tube assay which is an informative genotyping method designated for typing strains in both the conserved core and flexible accessory genome. However, this work strongly indicated that the population structure was more complex than previously reported [66].

The findings in this study are in support of a panmictic population structure for *P. aeruginosa* clinical and environmental isolates from both sides of the Mediterranean basin, punctuated by epidemic clones. We demonstrated an index of association of 0.35 for the entire population, and 0.07 when looking separately at the singleton sequence types. Under panmixis, linkage equilibrium will be observed and the I_A_^S^ approaches 0, whereas a clonal population will display an I_A_^S^ value that differs significantly from 0. More recently; by applying multilocus SNP typing on two unrelated strain collections, the index of association was consistently calculated in two independent studies to be 0.29 [43] and 0.31 [62]. This finding strongly indicates that the studied isolates of *P. aeruginosa* exhibited an epidemic non-clonal population structure.

The minimal spanning tree (MST) analysis shed further light on the role of epidemic clones, demonstrating a very important role of clonal complex 235. ST235 is the primary founder of this clonal complex, surrounded by 4 single locus variants, and isolates sharing this ST were found in all countries except Spain. Previous reports have linked this ST also to Spain. ST235 and also some other STs (17, 27,111, 155, 175, 179, 244 and 253) which have been encountered in different countries over several years were detected in this study, but not the worldwide dispersed ST277 [69]. Several new STs were detected (ST972-ST1002), demonstrating that the MLST database is still novel and continuously growing.

Remarkably, our dataset roughly disclosed CC235 as a highly successful clone widespread in the population. Maynard Smith et al. [33] first pointed out that the over-representation of closely related, high frequency (epidemic) clones in a sample will lead to an inflated estimate of clonality of such population as a whole. Oversampling of a single clone in an epidemic population structure will therefore result in an underestimation of homologous recombination rates. A wide spectrum of methods exist to estimate this ratio, however they vary in their ability to detect recombination [59], [70], [71]. For example, eBURST [55], [72] can estimate the ratio of r/m. We have rejected this test for two reasons, the first because this method has the disadvantage of scanning the clonal diversification between close relatives within clonal complexes, and could therefore produce inflated results if the role of recombination has increased in recent time. The second reason was that our dataset contained few larger clonal complexes; in fact only two dominant clonal complexes were detected.

ClonalFrame was developed to handle MLST data and is able to infer the rates at which mutation and recombination events occur over time, as well as the average size of recombination events [59]. By analysing our data with ClonalFrame we determined the r/m ratio to 8.4 (95% CI 4.7–13.7) demonstrating that homologous recombination has more impact on sequence evolution than mutation. Phylogenetic networks, such as the one constructed by the Neighbor-Net method, can represent the evolutionary relationships among recombining bacteria, as conflicting signals can be represented as a network instead of bifurcating tree. Using individual loci and concatenated sequences we found highly statistically significant recombination, supported also by visual inspection of the bushy network structure with complex parallelogram formation indicating a history of intragenic and intergenic recombination among housekeeping genes and responsible for the diversification of genotypes or sequences types. Here for *P. aeruginosa*, divergence among genotypes appeared to be mainly driven by recombination. The rate of homologous recombination within bacterial species can differ widely from one species to another [33], [73]. One of the striking features of *P. aeruginosa* is its capability to customize its genome to fit the needs for thriving in any actual and virtual environment [74]. This genomic reorganization is favored by the acquisition of blocks of genes through horizontal gene transfer for some strains and the deletions of specific chromosomal segments in others [19], [20], [22], [74]. As natural transformation is not encountered in *P. aeruginosa*, horizontal gene transfer appears to play a major role for the observed dynamic genome. As a consequence horizontal gene transfer enables *P. aeruginosa* to adapt to different habitats with acquisition of new traits without eliminating others, and hence the organism retains its ability to thrive in a wide range of environments [74].

The present study revealed that the clonal complex CC235 was strongly associated with O11 serotype strains from several countries, not restricted to particular clinical syndromes, and was also found in sea water and in the hospital environment (Table S1). Most of the CC235 strains featured virulence gene *exoU* and more than the half were MDR. Hence, CC235 is a successful epidemic clone associated with particular traits, but heterogeneous for others. This finding corroborates earlier observations that there is no correlation between *P. aeruginosa* clones and disease or habitats. [62], [63], [64], [75], [76].

The type III secretion system (TTSS) is considered as an important determinant of virulence for *P. aeruginosa* [5], [6], being present in some isolates and absent in others [77], and they are dispersed through the genome of *P. aeruginosa* [16]. Using TTSS, *P. aeruginosa* is able to deliver among others ExoS and ExoU inside eukaryotic cells. Exoenzyme S (*exoS*), a major cytotoxin involved in colonisation, invasion and dissemination of bacteria during infection, is regarded the most prevalent of the TTSS proteins [78]. Genes encoding these toxins are inconstant in *P. aeruginosa* isolates. In fact, *exoS* prevalence among cystic fibrosis patients is significantly higher than that in non-CF isolates [64], [79]. ExoU has been found to be associated with diverse infections [80]. In a mouse model of acute pneumonia, ExoU had the greatest impact on disease severity [81]. It has also been shown in one study that ExoU is substantially more cytotoxic than ExoS [82]. The prevalence of virulence genes in our collection corroborates previous reports [50]. Our study revealed that the genotype *exoU* was frequently associated with ST235-O11 isolates, and rarely occurred among O1, O10, O6 and O7 isolates. Earlier studies were partially in agreement with our findings [83], [84]. By comparing various genomes of various strains harbouring the *exoU* gene, Kulasekara et al. pointed out the evolutionary history of *exoU* locus, the mechanism including transposition of *exoU* determinants via horizontal transmission on plasmid followed by integration into different *P. aeruginosa* isolates [78]. Even though *exoS* and *exoU* are located in distinct loci [16], [85], the simultaneous carriage of both genes does usually not occur [22], [50], [84]. The mechanism of their incompatibility is still ambiguous. Our work revealed that few strains encoded both *exoS* and *exoU*, however this combination has been described earlier on rare occasions [64]. The explanation might be that *exoU* is transferred with genomic islands, and the acquisition of this gene through horizontal gene transfer may enhance colonisation and survival in different host environments [22]. Consequently, selective pressure probably acted as a driving force of these genomes in different environmental niches by mutual exclusion of *exoS* or *exoU* [22].

Most CC235 strains were shown to have distinct or unrelated PFGE patterns types and in some cases the similarity was below 80%. Similar observations have been made for e.g. the successful *E. coli* clone ST131, which can also exist in many PFGE-variants [86]. The diversity of PFGE-patterns in ST235 (data not shown) suggests the presence of microevolution within this sequence type.

By using MLST and PFGE, several independents studies focused on the underlying mechanisms of *P. aeruginosa* MDR. These studies provided evidence that ST235 is an international clone belonging to the BG11 complex that has been detected in Greece, Italy, Hungary, Poland, Sweden, Spain, France, Russia, USA, Japan [38], [47], [87], [88], [89], [90], [91], [92], [93], [94], [95], [96], [97] Singapore, and Brazil (http://pubmlst.org/paeruginosa/). Interestingly, the resistance determinants of ST235 isolates were mapped and examined and found to be associated with several acquired ß-lactamases: PER, OXA GES, VIM and IMP. ST235 has been linked to a variety of horizontally acquired genetic elements (integrons, transposons and plasmids) [93], [98]. Here we suggest an impact of microevolution discerned by a genetic capacity of ST235 isolates having undergone several genetic events giving rise to successful strains carrying out specific traits (MDR and O11).

The flexibility of the genome of ST235 clone and its trend to be widely dispersed in the world is reflected by the birth of several single locus variants (SLVs). Two novel SLVs were determined in our work. When, applying eBURST on MLST database (data not shown) we observed several groups, from which the CC235 is an interesting lineage that consisted of 29 STs (Figure 2; ST235 is primary founder surrounded by 19 SLVs). Strikingly and accordingly to the MLST database, CC235 displayed as a star like-tree with ST235 representing the parsimonious founder, it was indeed associated with the greatest number of SLVs (Figure 2). On the basis of these findings we argue that such a versatile genetic background enables clones such as ST235 clones to be successful and prevalent in many diverse habitats. However, we have likely shown a local polymorphism in CC235 strains which could influence their fitness in a drug resistance point of view. Also, ST235 clones were first described in the last few years, and the origin of them has not yet been defined.

In conclusion, this study confirms the hypothesis of a non-clonal epidemic population structure, and expands the current database to countries south of the Mediterranean basin. An important subtype was identified as CC235 O11 clone, often associated with *exoU* and multidrug-resistance and largely successful. Importanly, this clone plays a defining role in the dissemination of class A ESBLs and metallo-β-lactamases with potentially significant implications for public health. Lastly, the study demonstrates unequivocally that recombination is the most decisive factor for diversification of *P. aeruginosa* clones. Although the database includes non-MDR isolates, it would greatly benefit from a larger sample collection from different parts of the world. More work is necessary to further understand the phylogeny of *P. aeruginosa* and its population biology on a global level.

## Supporting Information

Figure S1 Neighbor-joining tree constructed using MEGA 4, showing relationships between the concatenated sequences of all *P. aeruginosa* STs (n =  70). Bootstrap values are indicated at corresponding nodes and STs at end of branches. Bar is 0.002 substitutions per site.
(TIFF)

Figure S2 SplitsTree networks for each individual locus of *Pseudomonas aeruginosa* housekeeping gene.
(TIFF)

Table S1 Database displaying the phenotypic and genotypic features of the studied strains: strains, sources, countries, allelic profiles, STs, serotypes, virulence genes, presence of multidrug-resistance, PFGE patterns, PFGE groups, clonal complexes. Representative isolates for PFGE-clusters that were subjected to MLST are highlighted in column 1.
(XLS)
