# Supplementary figures and images for: Population Structure of Pseudomonas aeruginosa from Five Mediterranean Countries: Evidence for Frequent Recombination and Epidemic Occurrence of CC235
Source: PLoS One. 2011 Oct 3;6(10):e25617. doi: 10.1371/journal.pone.0025617 (PMC3184967; doi:10.1371/journal.pone.0025617)

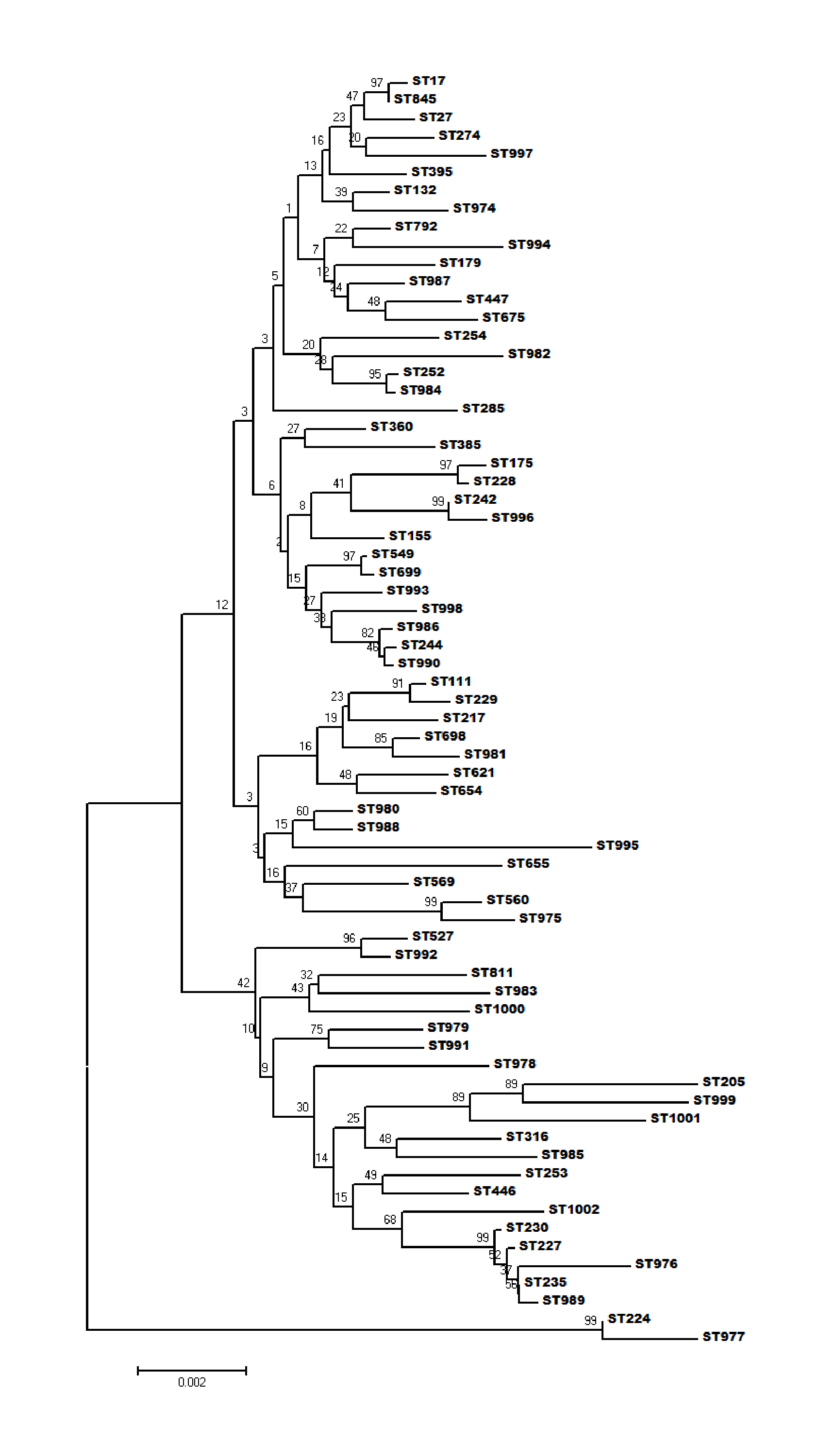

Supplement: Figure S1 — Neighbor-joining tree constructed using MEGA 4, showing relationships between the concatenated sequences of all P. aeruginosa STs (n = 70). Bootstrap values are indicated at corresponding nodes and STs at end of branches. Bar is 0.002 substitutions per site. (TIFF) [file pone.0025617.s001.tiff]

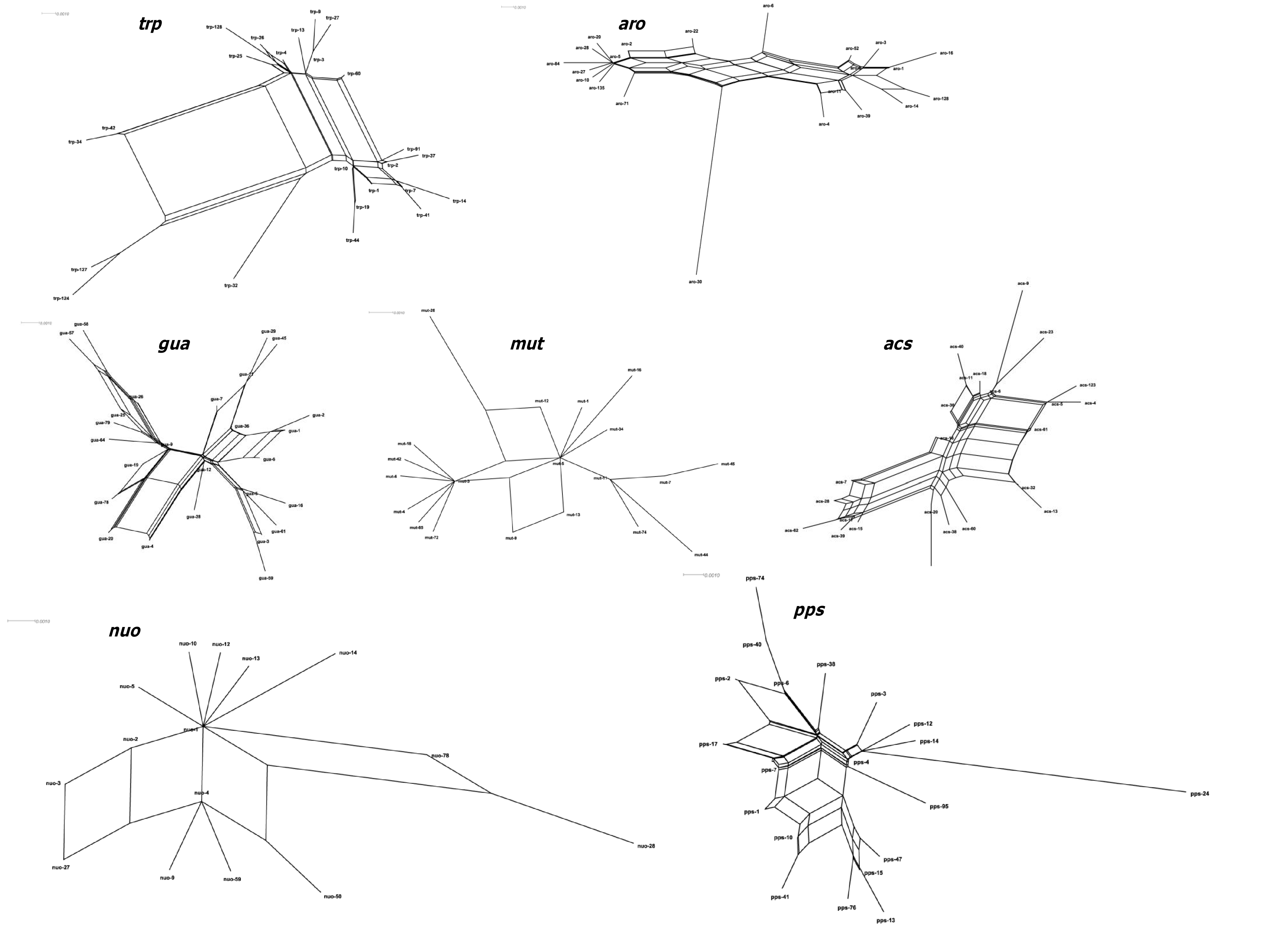

Supplement: Figure S2 — SplitsTree networks for each individual locus of Pseudomonas aeruginosa housekeeping gene. (TIFF) [file pone.0025617.s002.tiff]
